# Mitochondrial Role in Stemness and Differentiation of Hematopoietic Stem Cells

**DOI:** 10.1155/2019/4067162

**Published:** 2019-02-06

**Authors:** Luena Papa, Mansour Djedaini, Ronald Hoffman

**Affiliations:** Division of Hematology/Oncology, Tisch Cancer Institute, Icahn School of Medicine at Mount Sinai, New York, NY 10029, USA

## Abstract

Quiescent and self-renewing hematopoietic stem cells (HSCs) rely on glycolysis rather than on mitochondrial oxidative phosphorylation (OxPHOS) for energy production. HSC reliance on glycolysis is considered an adaptation to the hypoxic environment of the bone marrow (BM) and reflects the low energetic demands of HSCs. Metabolic rewiring from glycolysis to mitochondrial-based energy generation accompanies HSC differentiation and lineage commitment. Recent evidence, however, highlights that alterations in mitochondrial metabolism and activity are not simply passive consequences but active drivers of HSC fate decisions. Modulation of mitochondrial activity and metabolism is therefore critical for maintaining the self-renewal potential of primitive HSCs and might be beneficial for ex vivo expansion of transplantable HSCs. In this review, we emphasize recent advances in the emerging role of mitochondria in hematopoiesis, cellular reprograming, and HSC fate decisions.

## 1. Introduction

Hematopoiesis is a complex process that allows sustained production of each of the blood cell lineages throughout the lifespan of an individual. Vast numbers of adult mature blood cells are constantly generated from hematopoietic stem cells (HSCs) through a series of lineage-committed progenitor cells [1]. HSCs replenish the hematopoietic system with more committed progenitor and differentiated cells while they sustain long-term hematopoiesis. The balance between self-renewal (ability to generate themselves) and differentiation is central to blood cell homeostasis [2]. Cells in both states are characterized by distinct gene expression profiles, epigenetic landscapes, and developmental potentials [3]. Importantly, HSCs and committed progenitors as well as differentiated blood cells differ drastically in both their metabolic profiles and mitochondrial functions. Metabolic cues and mitochondrial DNA content, mass, and activity have been reported to vary within different stages of hematopoiesis [4–6].

Mitochondria are very complex and highly dynamic organelles. They are the major source of adenosine-5′-triphosphate (ATP) production through oxidative phosphorylation and sustained electron transport chain (ETC) activity. Mitochondrial OxPHOS is fueled by the tricarboxylic acid (TCA) cycle that converts pyruvate to acetyl-CoA. In addition, mitochondria serve as biosynthetic and signaling organelles [7]. The intermediates generated from the TCA cycle are essential for heme, amino acid, and nucleotide biosynthesis as well as for histone acetylation. Mitochondria are also the sites for fatty acid oxidation and steroid metabolism [8]. Besides their fundamental role in energy production and metabolism, mitochondria possess other important functions including calcium homeostasis, regulation of cellular and intracellular signaling, inflammation, and apoptosis, all of which are consistent with the notion that mitochondria act as a signaling organelle [9, 10]. These processes are impacted and regulated by reactive oxygen species (ROS), the by-products of OxPHOS activity. While mitochondrial OxPHOS activity is the most efficient pathway for energy production, glycolysis is another energy-generating pathway. During glycolysis, glucose is converted to pyruvate and then anaerobically to lactate. Importantly, glycolysis is preferentially utilized by HSCs [4, 11]. The potential benefit of the reduced need for mitochondrial functions in HSCs is the limitation of ROS levels. HSCs are particularly vulnerable to oxidative stress and high levels of ROS [12, 13]. Excessive ROS levels drive the exit of HSCs from quiescence, impair their multilineage differentiation capacity, and induce uncontrolled proliferation and sustained cumulative damage, ultimately leading to HSC exhaustion and loss of self-renewal potential [13–15].

Quiescent HSCs predominantly reside in regions of the bone marrow (BM) cavity termed niches, which provide a unique landscape with a low oxygen tension [16, 17]. As a consequence, the dependency of HSCs on glycolysis has been proposed to reflect their adaptation to low oxygen levels as well as their relatively low demands for energy [5, 12, 18]. During HSC differentiation and maturation, however, a rapid switch from glycolysis to mitochondrial OxPHOS and ATP generation occurs [4, 12, 19, 20]. This switch allows differentiating cells to meet their altered and higher metabolic and energy requirements associated with differentiation [11, 21]. An increase not only in mitochondrial activity but also in mitochondrial mass, membrane potential, and ROS levels accompanied by profound alterations in the mitochondrial ultrastructure characterizes the transition from quiescence to proliferation, from a primitive stem-like state to a differentiated state [12, 21–26]. By contrast, ex vivo reprograming of more differentiated cells into HSCs with the use of chromatin-modifying agents is associated with a reverse metabolic switch. In this review, we will discuss whether the alterations in the mitochondrial profile and function are simply passive consequences of changes in the status of HSCs or are in fact critical drivers of the transition from a stem cell to more differentiated cells. Moreover, we will review the recent evidence that emphasizes the role of mitochondria during reprograming of more committed cells to HSCs followed by their ex vivo expansion, a process that substantially increases the numbers of functional human HSCs that have potential therapeutic applications.

## 2. Mitochondrial Oxidative Phosphorylation versus Glycolysis in Determining HSC Fate Decisions

HSCs display unique properties and functions that distinguish them from more committed progenitors and mature blood cells. HSCs are predominantly quiescent, and their metabolic wiring and reliance on glycolysis are distinct from those of committed progenitors and the other cells in the BM that encompass primarily lineage-differentiated cells [4, 5, 27]. Unlike their progeny, HSCs accumulate high levels of 1,6-bisphosphate and other products of the final ATP-producing step of glycolysis. Such an increase in the levels of glycolytic by-products correlates with high pyruvate kinase (PDK) activity [22], which is dependent on hypoxia-inducible factor 1*α* (HIF1*α*). In turn, HIF1*α* drives and regulates a metabolic program that limits the engagement of the TCA cycle and sustains glycolysis as a main source of energy [28].

Enhanced mitochondrial activity is detrimental to the functional identity of HSCs and maintenance of their numbers. Loss of mitochondrial carrier homolog 2 (MTCH2) enhances OxPHOS activity and intracellular ROS levels, triggering the entry of HSCs into the cell cycle and loss of their self-renewal potential [29]. By contrast, lowering mitochondrial activity by chemically uncoupling the mitochondrial ETC sustains the self-renewal potential of HSCs in ex vivo cultures that normally induce differentiation [30].

In spite of the high preference for glycolysis, mitochondria in HSCs are not completely inactive. In fact, HSCs residing in the BM depend on mitochondrial activity and metabolism for their differentiation and survival. Suppression of OxPHOS activity in HSCs upon the loss of Ptpmt1, a PTEN-like mitochondrial phosphatase, impairs the early differentiation of HSCs and results in defective hematopoiesis [21]. Moreover, the mouse mutant, SDHD-ESR, that carries an inducible deletion of the *SdhD* gene, which encodes for one of the subunits of the mitochondrial complex II, is characterized by impaired survival of both HSCs and progenitors belonging to different lineages [31]. Of interest is also a recent study, which revealed that the complete disruption of mitochondrial respiration due to loss of the mitochondrial complex III subunit Rieske iron-sulfur protein (RISP) in fetal mouse HSCs leads to depletion of HSC numbers and their multilineage repopulation capacity [32]. Whereas RISP-null fetal HSCs have defects in their differentiation capacity, the RISP-null adult HSCs are characterized by loss of quiescence and entry into the cell cycle that is associated with lethality [32]. Collectively, these studies suggest that the self-renewing HSCs rely heavily, but not solely, on glycolysis, therefore emphasizing the importance of limited mitochondrial activity and metabolism in hematopoiesis and HSC fate decisions.

While the metabolic switch from glycolysis to mitochondrial OxPHOS activity and metabolism is required to meet the robust energy and metabolic demands imposed by differentiation, the precise mechanism underlying this switch remains elusive. It is likely that this metabolic rewiring is more complex than a simple switch from one form of energy production to another one. Instead, it might be the result of a series of events that occur before the onset of the well-known “metabolic switch.” Importantly, these events might engage mechanisms that not only impact but also act in concert with this “metabolic switch” to coordinate and control the balance between HSC self-renewal and differentiation.

## 3. Mitochondrial Mass and Membrane Potential in HSC Fate

**Figure 1:**
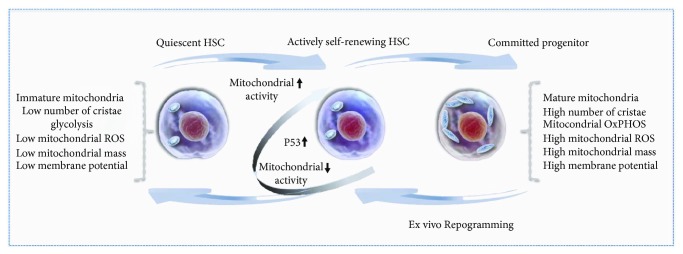
Mitochondrial regulation of HSCs. HSCs exhibit an immature mitochondrial network with globular mitochondria and primitive cristae. They rely heavily on glycolysis and display a low metabolic profile and OxPHOS activity accompanied by low mitochondrial ROS levels, mass, and membrane potential. During transition from quiescent to active cycling, HSCs increase their mitochondrial activity and potential to meet the increased demands of cycling cells for energy and metabolic bioproducts. During active cycling and divisions, suppression of the mitochondrial activity and activation of the p53 pathway are however required for HSCs to retain their self-renewing potential. By contrast, more committed progenitors display a mature mitochondrial network with the tubular mitochondria filled with a high number of regular cristae. Moreover, they exhibit high mitochondrial OxPHOS activity accompanied by high membrane potential, ROS generation, and mass. Ex vivo reprograming of more committed progenitors to actively dividing HSCs and their expansion are tightly linked to remodeling of a primitive mitochondrial network with low mitochondrial OxPHOS activity, increased glycolysis, and activation of the p53 pathway.

The role of mitochondria in HSC fate decisions and function is not merely limited to the metabolic switch but involves concomitant alterations of the mitochondrial features and properties. Distinctive mitochondrial membrane potential and mass between HSCs and cells at different stages of hematopoiesis have been reported. Indeed, both low mitochondrial activity and membrane potential mark the self-renewing murine and human HSCs [30, 33, 34] (Figure 1). Such HSCs possess significantly greater long-term multilineage reconstituting capacity in both primary and secondary NSG mice as compared to the same subpopulation of phenotypically defined HSCs that display a high mitochondrial membrane potential, indicative of mitochondrial bioenergetics [30]. Remarkably, these long-term HSCs have a lower mitochondrial mass as opposed to the more committed progenitors [30]. This evidence is consistent with findings indicating that a blockade in HSC differentiation by TSC1-mediated mTOR pathway inhibition is accompanied by a decrease in the mitochondrial mass [35]. By contrast, the loss of self-renewal capacity of HSCs due to lack of MTCH2 is related to an increase in both OxPHOS activity and mitochondrial size/volume [29].

The role and extent of mitochondrial membrane potential and mass on murine HSC self-renewal and commitment are similar to those observed during ex vivo studies performed with human umbilical cord blood-derived CD34^+^cells (UCB-CD34^+^) [34, 36]. The subpopulation of UCB-CD34^+^ cells enriched in cells with long-term repopulating potential exhibits low levels of both mitochondrial mass and membrane potential as opposed to more differentiated cells [26]. Interestingly, the loss of CD34 expression in human mobilized HSCs undergoing commitment correlates with an increase in the mitochondrial content [37]. Unlike differentiation, cellular reprograming of UCB-CD34^+^ cells into functional HSCs and acquisition of the CD90 phenotype triggered by treatment with a histone deacetylase inhibitor, valproic acid (VPA), are accompanied by a significant decline in mitochondrial mass and DNA content [36, 38]. Importantly, the reduction in mitochondrial mass is concomitant with a decrease in both mitochondrial OxPHOS activity and membrane potential [36] (Figure 1). Within the pool of ex vivo-expanded HSCs under normoxic conditions, cells expressing higher levels of both CD34 and CD90 exhibit lower mitochondrial mass compared to cells expressing low levels of both of these markers that phenotypically define functional HSCs [36].

Recently, the evidence that HSCs contain a low mitochondrial mass compared to progenitors and to mature cells has been challenged. A new study indicated that the mitochondrial mass in HSCs is underestimated due to artifacts caused by the efflux of MitoTracker Green, a commonly used dye to measure mitochondrial mass [39]. Intriguingly, another report revealed that both *in vivo* initiation and *in vitro* initiation of HSC division upon hematopoietic stress involve enhanced mitochondrial membrane potential and activity induced by increased Ca_2_^+^ flux [33] (Figure 1). While the amount of mitochondrial content in HSCs warrants further investigation, it is plausible that a parallel increase in both mitochondrial mass and potential may transiently precede the entry of HSCs to the cell cycle. To this end, however, it should be emphasized that following initiation of HSC divisions, retention of the self-renewing capacity of dividing HSCs requires suppression of the mitochondrial potential [33].

It is also important to reinforce at this point that even under normoxic condition HSCs display highly reduced mitochondrial activity as opposed to lineage-committed progenitors [4, 40]. In this regard, autophagy that is essential for HSC self-renewal potential acts primarily as a gatekeeper of metabolic activity [40–42]. HSCs undergo active autophagy, which limits the number of active mitochondria and therefore reduces not only the mitochondrial mass but also, and more importantly, the mitochondrial activity of HSCs [33, 40, 41]. Although this view has been challenged due to the heterogeneity of HSCs used in the majority of studies, a recent report has reinforced the emerging role of autophagy [41]. In fact, the self-renewal potential of purified Tie2^+^ HSCs that were identified to be at the top of the HSC hierarchy by both single-cell analysis and cell transplantation depends on activation of autophagy and particularly mitophagy [41].

It appears that the activation of autophagy is not balanced by enhanced mitochondrial biogenesis. In support of this notion is the evidence that mTOR pathway inhibition contributes to HSC quiescence not only by promoting autophagy but also by repressing mitochondrial biogenesis [35, 43–47]. In fact, the maintenance of HSC self-renewal relies on the repression of mitochondrial biogenesis and metabolic activity [25, 35, 48]. Conversely, transition of HSCs from quiescence to active proliferation is intrinsically related to enhanced mitochondrial biogenesis [49]. Mitochondrial metabolic fitness during this transition is tightly monitored by the mitochondrial unfolded protein response (UPR^mt^), which is currently emerging as one of the main mitochondrial quality control mechanisms for HSC self-renewal. Interestingly, one of the key elements of the UPR^mt^, SIRT7, protects the HSC pool challenged by stress by suppressing mitochondrial biogenesis [50]. This evidence underlines the remarkable ability of HSCs to activate multiple mechanisms and tightly control mitochondrial metabolic activity, which appears to be not simply a hallmark but rather a critical determinant of HSC maintenance and functional identity.

## 4. Multifaceted ROS and Their Role in HSC Fate

HSC fate decision with regard to self-renewal or commitment is monitored and regulated by ROS, a by-product of the bioenergetic metabolism. Although critical for physiological processes including activation of signal transduction pathways and fighting pathogens, excessive ROS can impair cellular functions by causing oxidative damage to lipids, proteins, RNA, and DNA. While the mitochondria are the major sources of ROS generation, they are also the main targets of ROS leading to vicious cycles of mitochondrial damage and energetic catastrophe. As discussed above, such damage and a complete failure in mitochondrial activity eventually lead to HSC exhaustion and impaired differentiation. Excessive ROS contribute to HSC aging and senescence, and at even higher levels, ROS induce HSC cell death [51].

Although evidence points towards the need for a tight control of ROS levels to prevent tissue damage and cell death, it is becoming clear that ROS might function as a rheostat that regulates cell fate decisions. At low levels, ROS maintain quiescence and the long-term repopulating capacity of HSCs [46]. At moderate levels, however, ROS act as second messengers and govern changes in cell fate. A limited elevation in ROS levels is necessary to drive HSC differentiation [14, 52]. Moderate levels of ROS are needed for hematopoiesis during both embryonic development and adult homeostasis [53]. Enhanced ROS levels are also required for proliferation of HSCs and progenitor cells during recovery from bone marrow injuries [54]. Thus, at different concentrations, ROS exert different roles. In fact, differential modulation of ROS levels by MCL-1 and BID, both members of the BCL-2 family of apoptotic proteins, can lead to HSC self-renewal, hematopoietic differentiation, or cell death [55, 56]. Suppression of high mitochondrial ROS levels by ATM-mediated BID phosphorylation regulates HSC self-renewal and quiescence. In a steady state, loss of BID phosphorylation and its increased association with mitochondria induce ROS generation at levels that are sufficient to drive HSCs into active proliferation and cell cycle progression, but not to cell death [55]. However, upon stress, loss of BID phosphorylation results in an immense generation of ROS ultimately causing exhaustion of the HSC pool [55, 56]. These findings are consistent with a report indicating that low levels of ROS can be used to enrich for highly primitive and quiescent HSCs capable of establishing long-term engraftment in murine models [14]. Conversely, HSCs with high ROS levels demonstrate remarkable exhaustion following serial transplantations [14, 55, 57]. Thus, these studies collectively address the intriguing puzzle of the need for low ROS levels in the maintenance of HSC self-renewal and integrity, but the absolute necessity for a limited ROS elevation was during hematopoietic development and stress.

### 4.1. Balanced ROS Regulation in HSCs by Coordinated Activity of Redox Signaling, Metabolism, and Epigenome

Regulation of ROS levels in HSCs is highly complex and involves regulation of both HSC metabolic activity and their antioxidant defense mechanisms [5, 58, 59]. Several lines of evidence indicate that MEIS1 (myeloid ecotropic viral integration site 1 homolog) regulates both of these processes by activating HIF1*α* and HIF2*α* [28, 58, 59], both of which drive cellular metabolism towards anaerobic glycolysis instead of mitochondrial respiration [4, 28, 46, 60]. Although the role of HIF1*α* and HIF2*α* in HSC function [61, 62] has been recently challenged, HSC maintenance by MEIS1 and its role in limiting oxidative stress have been well established using numerous mouse models [58, 59, 63, 64]. In addition to MEIS1, several other molecular pathways have been reported to act as central hubs that integrate metabolism with redox signaling and epigenetic modification. These pathways, which include class O of forkhead box (FoxOs) family proteins, sirtuin family members (SIRTs), p53 (TP53), the nuclear factor-kappa B (NF-*κ*B), mTOR, and epigenetic regulators such as histone deacetylases (HDACs), are tightly interconnected. Together, they control, in a coordinated manner, the equilibrium between quiescence, active cycling, and differentiation of HSCs [15, 22, 65, 66]. Of great interest in this regard are the sirtuins, which have emerged as stress-responsive enzymes that govern cellular adaptations by altering the acetylome [67]. SIRT1 contributes to the maintenance of HSCs by limiting high levels of ROS through FOXO activation and decreased p53 activity [68–74]. It is likely that following metabolic stress, SIRT1 mitigates high ROS levels in HSCs by also activating a rapid induction of FOXO3A-dependent autophagy [75, 76]. In addition, SIRT1 retains both the genomic landscape and the epigenetic landscape of adult HSCs by promoting polycomb-specific repressive histone modification [77]. Notably, polycomb proteins, in particular BMI-1, which is a master epigenetic regulator of HSC self-renewal and fate, control mitochondrial ROS generation, further linking ROS with the epigenome and the fate of HSCs [78, 79].

**Figure 2:**
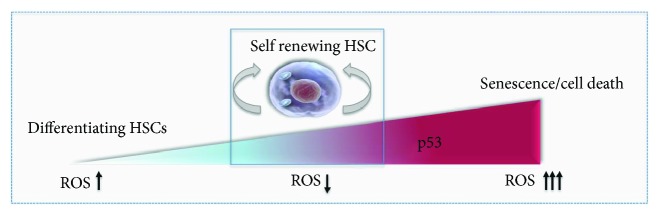
Effect of different levels of p53 in HSCs. Adequate p53 level and activity regulate the quiescent and self-renewing potential of HSCs by reducing ROS levels (boxed area). Lack of p53 impairs the quiescent state and self-renewing potential of HSCs and compromises the functional fitness of HSCs. Decreased p53 expression levels and activity promote differentiation of HSCs. Upon high levels of oxidative stress and ROS, increased p53 activity leads to senescence or cell death.

The orchestration of the redox status of HSCs is monitored by the antioxidant defense mechanism, which relies on the activity of the scavenger antioxidant enzymes such as MnSOD. Sirtuin family proteins, primarily SIRT3, are required to retain the regenerative capacity of aged HSCs and to limit ROS production by enhancing MnSOD activity [80]. The long-term reconstitution capacity and premature aging of HSCs are also impacted by deficiencies in other redox sensors including thioredoxin-interacting protein (TXNIP) [65, 81]. The capability of TXNIP to regulate the aging of HSCs is attributed to its direct interaction and inhibition of the p38 MAPK pathway [81]. Whereas p38 activation by ROS limits the lifespan of HSCs [52], inhibition of p38 restores the long-term reconstitution capacity of HSCs. Moreover, p38 activation has been shown to deplete human HSCs and to be associated with the development of aplastic anemia in man [14, 52, 82]. In addition, TXNIP acts to retain the HSC pool by switching the function of p53 from serving as a prooxidant to an antioxidant [65]. Indeed, p53 is required for the maintenance of quiescent HSCs [83], and loss of p53 impairs the long-term repopulating capacity and functional identity of HSCs in serial transplantation assays [84] (Figure 2). Remarkably, a recent report demonstrated that suppression of Ca_2_^+^-mediated mitochondrial functions contributes to the maintenance of self-renewing murine HSCs during cell divisions through upregulation of p53-related genes [33]. At the same time, another study by our group revealed that the antioxidant activity of p53 is essential for the successful ex vivo reprograming and expansion of primitive HSCs from more committed UCB-CD34^+^cells with VPA treatment [36]. To prevent excessive ROS generation, cells undergoing reprograming with VPA treatment mount a cellular antioxidant defense that relies on the activity of the p53-MnSOD axis. Notably, this defense mechanism acts in concert with a remodeled primitive mitochondrial network, which exhibits reduced OxPHOS activity [36]. Consistent with this decrease is the suppression of p38 activity as well as the upregulation of MEIS1 [36]. Together, these events underscore the array of coordinated mechanisms that control ROS levels and limit mitochondrial functions required for cellular reprograming of human functional HSCs and their ex vivo expansion. Collectively, these studies point towards the complex and unique regulation of HSC fate decisions dictated by mitochondrial function and the dynamic changes in ROS levels.

## 5. Mitochondrial Dynamics in HSCs

Mitochondrial bioenergetics and structure are tightly linked. Mitochondrial dynamics, including the modulation in mitochondrial ultrastructure, has been suggested to play a fundamental role in mitochondrial metabolism and, therefore, in determining stem cell fate [85, 86]. Emerging evidence indicates that massive remodeling of the mitochondria and particularly cristae, which are highly dynamic compartments where the OxPHOS complexes reside, might reflect changes in the energetic state of the cell [85, 87]. Consistent with a reliance on glycolysis and limited mitochondrial OxPHOS activity, human HSCs as well as human and mouse embryonic stem cells (ESCs) contain rare mitochondria with immature, globular morphology and poorly developed cristae [37, 88–90]. During differentiation, mitochondrial maturation results in the appearance of more mature, elongated, and tubular mitochondria with well-developed cristae that reflects increased mitochondrial activity [91–93]. Indeed, impairment of HSC long-term reconstitution capacity due to the loss of imprinting at the Dlk1-glt2 locus is linked to enhanced mitochondrial activity and ROS levels, as well as increased folds of cristae [25]. Consistent with this, the loss of MITCH2, which primes mitochondrial OxPHOS and increases mitochondrial size/volume, results in accelerated differentiation to progenitor cells, loss of quiescence, and eventual HSC exhaustion [29].

Mitochondrial dynamics and morphology are orchestrated by the mitochondrial fission/fusion machinery that relies on the activity of shaping proteins such as optic atrophy (OPA1), mitofusin-1 and mitofusin-2 (Mfn1 and Mfn2), and dynamin-related protein 1 (Drp1). Indeed, the shape of mitochondria is continuously defined by antagonistic and balanced activities of fusion and fission proteins [94]. Aberrations in the mitochondrial fission/fusion machinery accompanied by a shift towards fusion favor the generation of abundant, large, and highly interconnected mitochondrial networks that are beneficial to metabolically active cells [85]. Such enlargement of mitochondria is due to decreased mitochondrial translocation of Drp-1, which is a master regulator of mitochondrial fragmentation [94]. Interestingly, the increase in mitochondrial size and the impairment of HSC function and numbers are due to loss of MITCH2, which is associated with a decreased association of Drp1 with mitochondria [29]. However, another study demonstrated that the fusion protein, Mfn2, which increases the buffering of intracellular Ca2^+^, is also required for the maintenance of the lymphoid potential of HSCs, suggesting that mitochondrial dynamics influences HSC fate through numerous mechanisms [95].

The current understanding of mitochondrial architecture in HSCs and the role of fission/fusion in retaining of HSC self-renewal potential is very limited. A better understanding has been achieved by studies performed during reprograming of somatic cells into induced pluripotent stem cells (iPSs). During reprograming to iPCs, mitochondria undergo significant remodeling accompanied by an early wave of mitochondrial fragmentation due to increased fission and Drp1 activity [96]. In fact, mitochondrial fragmentation is required during the reprograming process to a pluripotent state [91, 92, 96–98]. Consistent with this, a study by our group revealed that cellular reprograming of HSCs from more committed UCB-CD34^+^ cells is accompanied by a profound remodeling of the mitochondrial network comprised of morphologically small and globular mitochondria [36]. This cellular reprograming encompasses not only mitochondrial morphological changes but also a shift from OxPHOS activity to glycolysis (Figure 1). While the mechanism for such remodeling during HSC reprograming remains unknown, these data underlie the tight linkage between the mitochondrial ultrastructure and ROS generation, membrane potential, and mass in human primitive HSCs.

## 6. Conclusion and Perspectives

HSC reliance on glycolysis has been perceived as an adaptation to the hypoxic niche of the BM until now. Current evidence emphasized in this review, however, suggests that hypoxia might not be the only cause of limited mitochondrial metabolism in HSCs. In fact, numerous studies highlight the plasticity of mitochondria and their profound role in controlling the self-renewal and maintenance of HSCs. Important cues regarding the mechanisms and roles of mitochondria as drivers of HSC fate might be applied to improve efforts aimed at manipulation and ex vivo expansion of HSCs from UCBs. Ex vivo-expanded HSCs have potential therapeutic benefits in regenerative medicine to be used as allogeneic grafts for transplantation and/or gene therapy for monogenetic inherited blood disorders.

The use of UCBs for bone marrow transplantation is restricted due to the limited number of HSCs within a single unit. Several ex vivo strategies including the aryl hydrocarbon receptor antagonist (SR-1), pyrimidoindole derivative (UM-171), and VPA are currently utilized to overcome this limitation and expand to a great degree the numbers of transplantable HSCs [36, 38, 99, 100].

Ex vivo HSC cultures have been reported to induce stress. Such stress can impact mitochondrial function and activity, compromising therefore the characteristics of primary and clinically relevant HSCs. Overcoming this stress by limiting mitochondrial potential and activity presents an exciting target by which to expand the numbers of cycling HSCs while they retain their self-renewal and primitive characteristics. Remarkably, VPA treatment in ex vivo cultures has been reported to trigger both the acquisition and retention of a transcriptome and primitive mitochondrial profile with low activity, characteristic of primary functional HSCs [36, 38]. Both the acquisition and maintenance of a primitive HSC status were influenced by the antioxidant effect of the p53 pathway, which interestingly is reported to be enriched and activated during HSC self-renewing divisions [33, 36] (Figures 1 and 2). Thus, manipulation of both mitochondrial activity and the antioxidant p53 activity open new perspectives for the robust expansion of the self-renewing HSCs in ex vivo cultures. Such manipulations might be also beneficial for autologous HSC gene therapy and have the potential to overcome the loss of functional HSCs associated with gene editing. An adequate regulation of p53 activity and level also has the potential to preserve the functional fitness of HSCs with a youthful gene expression signature, both of which are lost during aging [101, 102]. Certainly, further understanding of the molecular mechanisms orchestrated by mitochondria in maintaining primary HSCs and determining their fate decisions will be essential to accelerate their application in regenerative medicine and transplantation settings.
